# Thermal rejuvenation in metallic glasses

**DOI:** 10.1080/14686996.2017.1280369

**Published:** 2017-02-20

**Authors:** Junji Saida, Rui Yamada, Masato Wakeda, Shigenobu Ogata

**Affiliations:** ^a^ Frontier Research Institute for Interdisciplinary Sciences (FRIS), Tohoku University, Sendai, Japan; ^b^ Graduate School of Engineering Science, Osaka University, Toyonaka, Japan; ^c^ Center for Elements Strategy Initiative for Structural Materials (ESISM), Kyoto University, Kyoto, Japan

**Keywords:** Metallic glass, rejuvenation, relaxation, molecular dynamics simulation, mechanical property, local structure, 10 Engineering and Structural materials, 106 Metallic materials, 302 Crystallization / Heat treatment / Crystal growth, 400 Modeling / Simulations

## Abstract

Structural rejuvenation in metallic glasses by a thermal process (i.e. through recovery annealing) was investigated experimentally and theoretically for various alloy compositions. An increase in the potential energy, a decrease in the density, and a change in the local structure as well as mechanical softening were observed after thermal rejuvenation. Two parameters, one related to the annealing temperature, *T*_a_/*T*_g_, and the other related to the cooling rate during the recovery annealing process, *V*_c_/*V*_i_, were proposed to evaluate the rejuvenation phenomena. A rejuvenation map was constructed using these two parameters. Since the thermal history of metallic glasses is reset above 1.2*T*_g_, accompanied by a change in the local structure, it is essential that the condition of *T*_a_/*T*_g_ ≥ 1.2 is satisfied during annealing. The glassy structure transforms into a more disordered state with the decomposition of icosahedral short-range order within this temperature range. Therefore, a new glassy structure (rejuvenation) depending on the subsequent quenching rate is generated. Partial rejuvenation also occurs in a Zr_55_Al_10_Ni_5_Cu_30_ bulk metallic glass when annealing is performed at a low temperature (*T*_a_/*T*_g_ ~ 1.07) followed by rapid cooling. This behavior probably originates from disordering in the weakly bonded (loosely packed) region. This study provides a novel approach to improving the mechanical properties of metallic glasses by controlling their glassy structure.

## Graphical abstract

**Figure:**
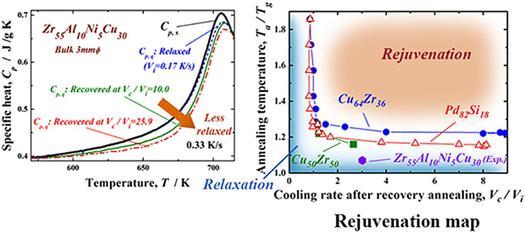

## 1. Introduction

Metallic glasses have been applied in industry owing to their attractive mechanical, physical, and chemical properties [1–3], which originate from the intrinsic random atomic configuration. With respect to this random structure, relaxation phenomena have been recognized as important subjects in fundamental and applied research [4]. It is well known that structural relaxation of metallic glass proceeds by annealing around or below the glass transition temperature, *T*_g_. A number of studies on the local structure [5,6], thermodynamics [7–10], free volume kinetics [11–13] and crystallization [14,15] associated with a relaxation state in metallic glasses have been reported. In application fields, relaxation is undesirable since it generally results in brittleness in many glassy alloy systems. Thus, controlling the relaxation state of metallic glasses is very important for improving their mechanical properties. However, it has been regarded that the recovery of a less relaxed state (i.e. rejuvenation) is very difficult, since the relaxation process is a non-reversible reaction. Therefore, rejuvenation has been induced only by introducing severe plastic deformation through processes such as shot-peening, cold rolling, or high-pressure torsion [16–18], which result in a forced transition of the glassy solid into an inhomogeneous non-equilibrium state. That is, partial rejuvenation occurs and one cannot generally obtain homogeneously rejuvenated bulk metallic glasses (BMGs) using these methods.

The inhomogeneous local structure of metallic glasses, which consists of a weakly bonded region (or soft, loosely packed region) and a strongly bonded region (or hard, closely packed region), has been investigated widely and has been found to be strongly related to several essential processes, such as relaxation, transformation, and deformation [4,19–23]. The relaxation behavior in the low-temperature region (for example, β-relaxation) as well as nucleation of the potential shear transformation zones takes place in the weakly bonded region, as the local atomic motions correlated with these events are readily activated there. A new concept for controlling the relaxation state of metallic glasses using such structural heterogeneity has been proposed. Ketov et al. [24] suggested a novel approach for rejuvenating metallic glasses by a non-affine strain introduced by thermal cycling between room and liquid nitrogen temperatures. The introduction of the non-affine thermal strain is attributed to an intrinsic non-uniformity (heterogeneity) of the glassy structure at the short- and/or medium-range scale, that is, owing to the non-uniform coefficient of thermal expansion during thermal cycling. Considering the mechanism of the non-affine thermal strain induced by the above process, this effect would be more pronounced in fragile glasses than in strong glasses, because of the improved heterogeneity of fragile glasses [25].

We have been studying relaxation phenomena in metallic glasses and previously reported that the relaxation state is determined by the cooling rate in the temperature region just above *T*_g_ (~ 1.2*T*_g_), not in the high-temperature region just below the melting temperature [26]. In this low-temperature region (~ 1.2*T*_g_), the relaxation time might be in the order of 10^−1^ to 10^−3^ s [27], which is equivalent or long enough to compare to the actual cooling rate using conventional Cu mold casting. This suggests that it should be possible to control the relaxation state in metallic glasses using a conventional thermal process. In other words, it should be possible to rejuvenate a relaxed glass by low-temperature annealing and subsequent rapid cooling. Rejuvenation has been widely investigated in correlation with the mechanical properties of polymer glasses [28–30]; however, preliminary work on recovering the ductility of fully relaxed Zr-Ti-Cu-Ni-Be BMGs by annealing under appropriate conditions at temperatures higher than *T*_g_ has been performed by Kumar et al. [31], in the first report on the possibility of rejuvenation of metallic glass by a thermal process. They clarified that the ductility can be recovered in brittle metallic glasses in the almost fully relaxed state by post-annealing at a temperature above *T*_g_ for a short duration followed by water quenching. However, they did not mention the annealing and cooling conditions necessary for rejuvenation. In a previous study, we also observed an increase in the enthalpy of relaxation, as calculated from calorimetric measurements, in a Zr-Al-Ni-Cu BMG after annealing at temperatures higher than *T*_g_ followed by cooling at a rate in the order of a few kelvins per second [32]. These results demonstrate a rejuvenation induced by a simple process of annealing and cooling of the glassy structure. Here, mechanical softening, indicated by decreasing hardness and Young’s modulus, are simultaneously observed along with the rejuvenation. A molecular dynamics (MD) simulation study has clarified that a new relaxation state can be induced in metallic glasses above *T*_g_, in which it strongly depends on the cooling rate after a metallic glass is heated [33]. The critical temperature for inducing the new relaxation state in this annealing condition is approximately 1.16*T*_g_ for Cu_50_Zr_50_. On the basis of these studies, it has been suggested that the annealing temperature and cooling rate are the dominant factors in rejuvenation. In the present study, we further investigated the mechanism and conditions for rejuvenation of several metallic glasses with the changes in the properties using the normalized parameters of the annealing temperature and cooling rate. Despite the importance of controlling relaxation in fundamental as well as application fields, only a limited range of conditions has been proposed for rejuvenation for each alloy composition. However, since rejuvenation can be regarded as a universal phenomenon in metallic glasses, it is essential to establish its mechanism as well as the common conditions. This study also aimed to propose a new concept of rejuvenation by controlling the glassy structure at the atomic level for a useful information for the applications of BMGs.

## 2. Experimental procedure

**Figure 1.**
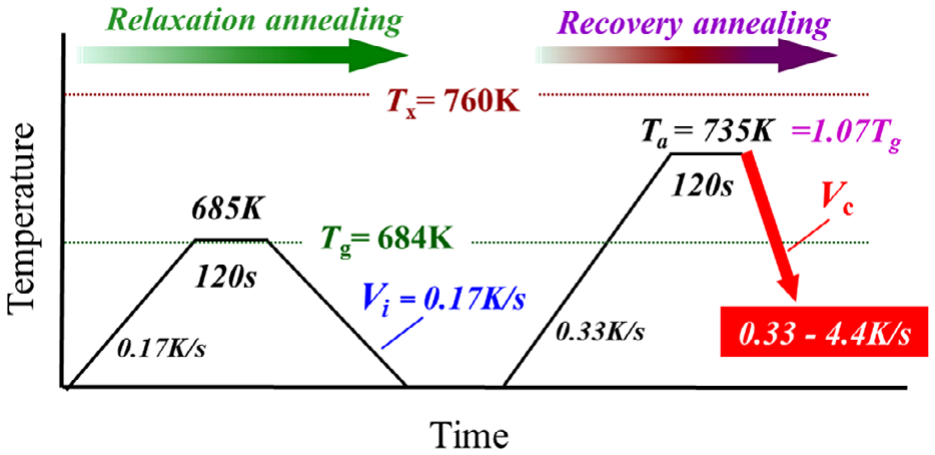
Annealing conditions for rejuvenation of the Zr_55_Al_10_Ni_5_Cu_30_ alloy.

Cylindrical bulk glassy samples 3 mm in diameter were produced by the Cu mold casting technique using a master ingot of Zr_55_Al_10_Ni_5_Cu_30_ alloy. The cooling rate near *T*_g_ was estimated to be 30 K s^–1^.[26] The measured glass transition (*T*_g_) and crystallization (*T*_x_) temperatures at a heating rate of 0.33 K s^–1^ are 684 and 760 K, respectively. Disc samples with a thickness of 0.5 mm were prepared by slicing the glassy rods. Figure 1 shows the annealing conditions used in this study. All the disc samples were relaxed by annealing for 120 s at 685 K at heating and cooling rates of 0.17 K s^–1^ (relaxation annealing) under a purified Ar flow using differential scanning calorimetry (DSC) (Perkin-Elmer Pyris Diamond DSC, Shelton, Connecticut, USA). The relaxation state should be almost the same in each disc sample after relaxation annealing. Here, we define the cooling rate of 0.17 K s^–1^ as the initial cooling rate, *V*_i_, of the starting material for recovery annealing. The relaxed disc samples were annealed for 120 s at *T*_a_ = 735 K (1.07*T*_g_) at a heating rate of 0.33 K s^–1^ and subsequently cooled at various rates *V*_c_ of 0.33–4.4 K s^–1^ (recovery annealing). The cooling rates were determined experimentally from a measured temperature curve just above *T*_g_ obtained by DSC. In this study, we examined the properties of the rejuvenated metallic glass samples using the normalized recovery annealing temperature, *T*_a_/*T*_g_, and the cooling rate, *V*_c_/*V*_i_, for a universal evaluation, as both parameters dominantly affect the rejuvenation process [33]. The structures of the samples after recovery annealing were analyzed by X-ray diffraction (XRD, Cu-Kα, 40 kV, 40 mA) and transmission electron microscopy (TEM) with an accelerating voltage of 200 kV. The relaxation states of the glassy structures were evaluated using the enthalpy of relaxation, ∆*H*_relax_, which is defined as follows:[34]

(1) $$\Delta H_{\text{relax}}=\int_{\;\text{RT}}^{723K}\Delta C\text{p d}T,$$

where Δ*C*_p_ = *C*_p,s_ – *C*_p,q_. Further, *C*_p,s_ and *C*_p,q_ are the specific heats of the sample reheated up to 723 K and in the as-recovery annealed state, respectively. The specific heats were measured at the heating and cooling rates of 0.33 K s^–1^ under a purified Ar flow. The holding time at 723 K was 120 s. ∆*H*_relax_ decreases with relaxation of the glassy structure; that is, a less relaxed (unrelaxed) glass has a larger ∆*H*_relax_ value. The mechanical properties of the recovery-annealed samples were evaluated using a micro Vickers hardness tester at a load of 20 g with at least 10 measurements for each sample. The elastic modulus was measured using a nanoindentation tester in the load control mode using a maximum load of 20 mN under a constant rate of 2 mN s^–1^. The holding time at the peak load was 1 s. Measurements were performed 10 times for each sample to verify the accuracy and scatter of the data. The density of the glassy samples was measured by the Archimedean method using *n*-tridecane as the working fluid. A glassy disc 5 mm in diameter was used for the density measurement to ensure the accuracy of the value. The internal loss of a Zr_55_Al_10_Ni_5_Cu_30_ metallic glassy ribbon was measured by tensile dynamic mechanical analysis (DMA) at a heating rate of 8.3 × 10^−2^ K s^–1^ and a frequency of 1 Hz.

MD simulations were also performed to investigate the rejuvenation process. We used the embedded atom model (EAM) potential developed for the Cu_64_Zr_36_ and Pd_82_Si_18_ metallic glasses [35]. The total number of atoms was 50,000, and three-dimensional periodic boundary conditions were applied. The MD time step was set to 1 fs. The NPT ensemble was employed for which the temperature and pressure are controlled by the Nosé-Hoover [36] and Parrinello-Rahman methods [37], respectively. The glassy mode was constructed via melt-quenching at a constant cooling rate of 10^12^ K s^–1^ (*V*_i_) from 2000 K to 0 K. *T*_g_ was determined to be 700 K for both alloys from the kink in the volume–temperature curve. The constructed glassy alloys were annealed by subsequent thermal loading (recovery annealing). The as-quenched metallic glasses were reheated at a rate of 10^13^ K s^–1^ to various temperatures, *T*_a_ above *T*_g_. After the metallic glasses were isothermally held for 2 ns, they were quenched at various cooling rates, *V*_c_ of 10^11^ to 10^13^ K s^–1^. The density and potential energy during the recovery annealing process were evaluated. The changes in the number of icosahedral atomic configurations and Young’s modulus of Cu_64_Zr_36_ were also calculated.

## 3. Results

### 3.1. Fundamental properties of thermally rejuvenated metallic glasses

**Figure 2.**
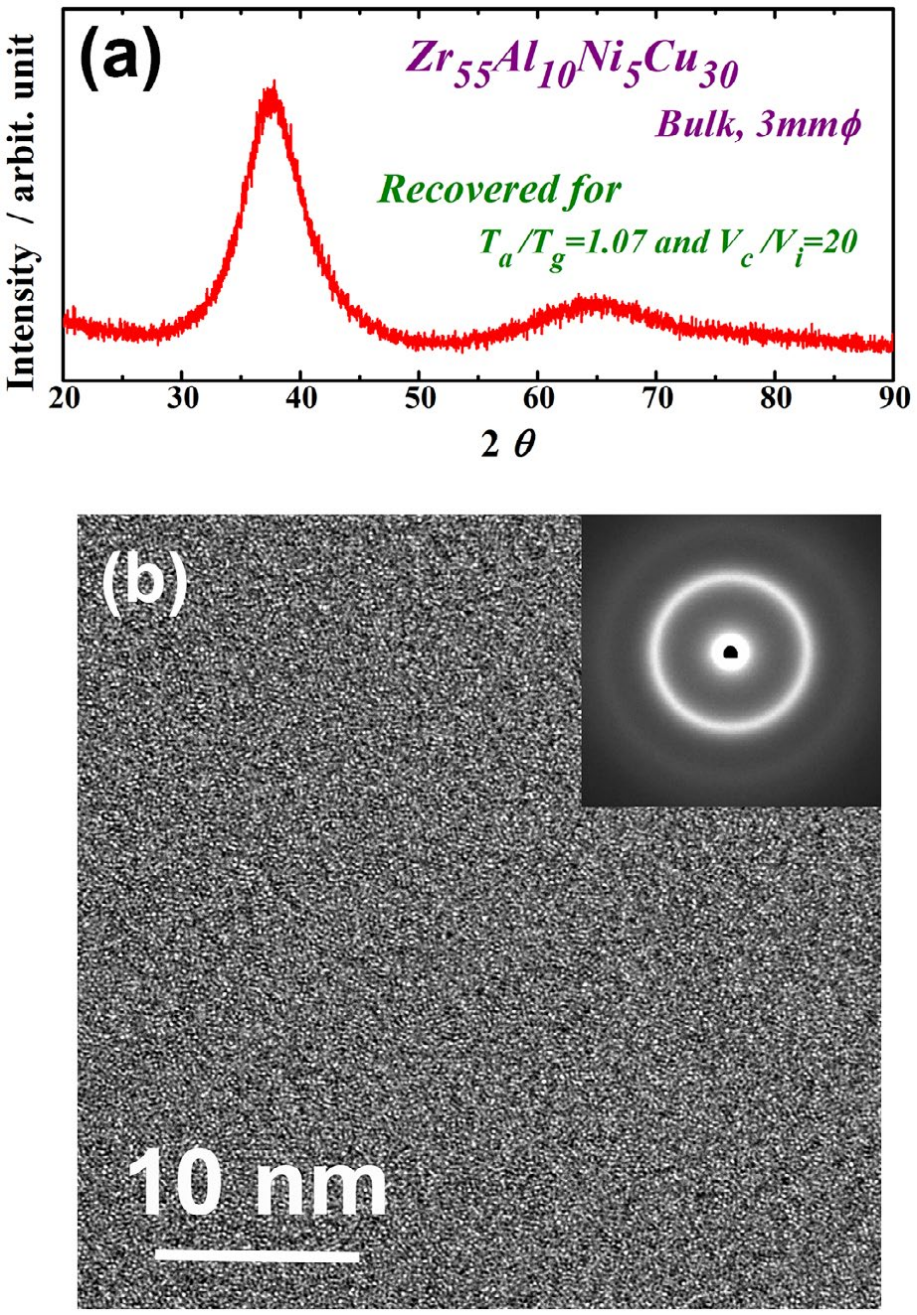
(a) XRD pattern and (b) high-resolution TEM image and selected-area electron diffraction pattern of the Zr_55_Al_10_Ni_5_Cu_30_ disc sample recovery-annealed at a temperature *T*_a_/*T*_g_ of 1.07 and cooling rate *V*_c_/*V*_i_ of 20.

**Figure 3.**
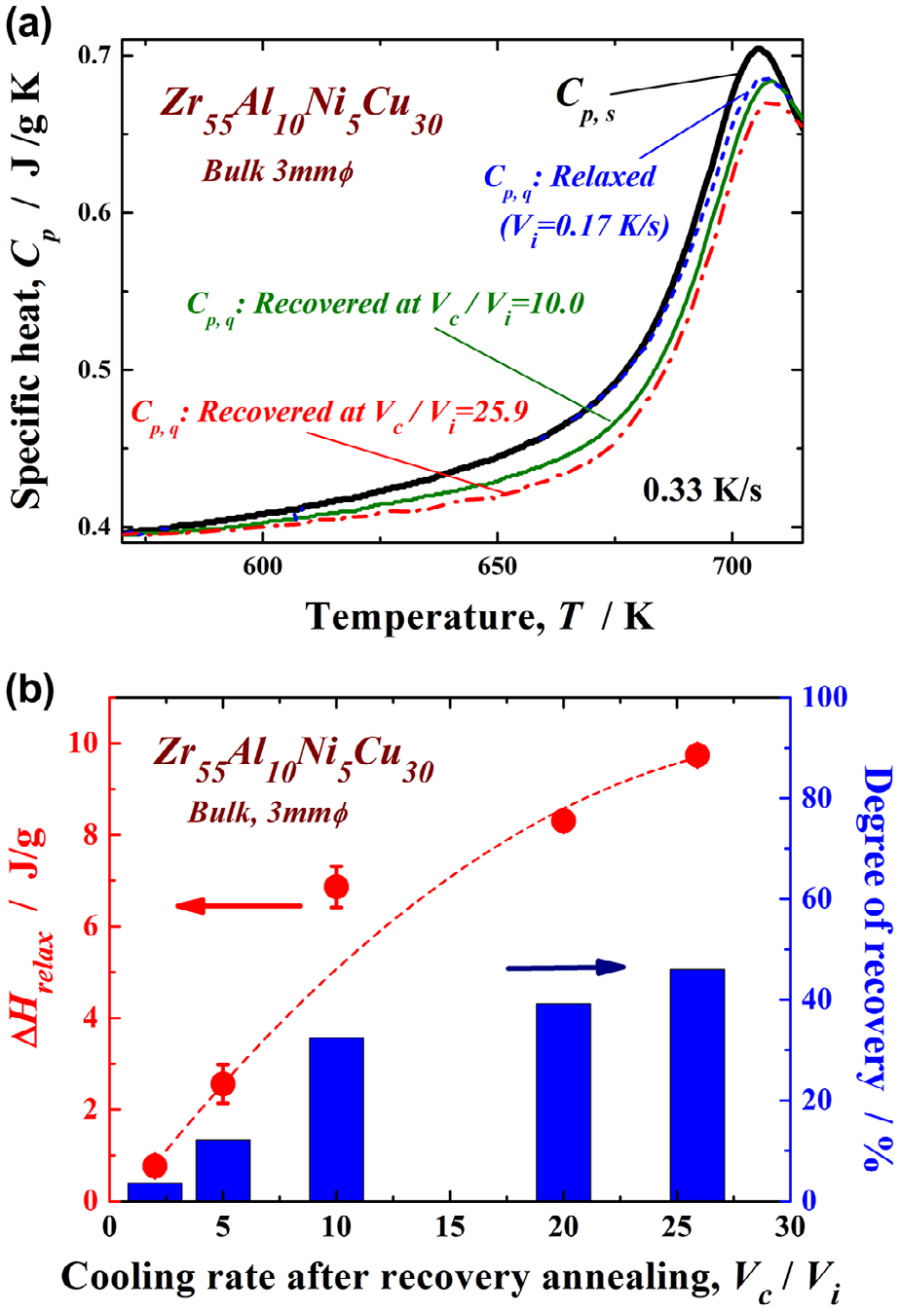
(a) Specific heat curves of the Zr_55_Al_10_Ni_5_Cu_30_ metallic glass in the relaxed and recovered (*V*_c_/*V*_i_ = 10.0 and 25.9 at *T*_a_/*T*_g_ = 1.07) states. (b) Change in ∆*H*_relax_ with *V*_c_/*V*_i_. The degree of recovery evaluated using$\Delta H_{\text{relax}}/\Delta H_{\text{relax}}^{\text{as - cast}}\;$ is also shown.

**Figure 4.**
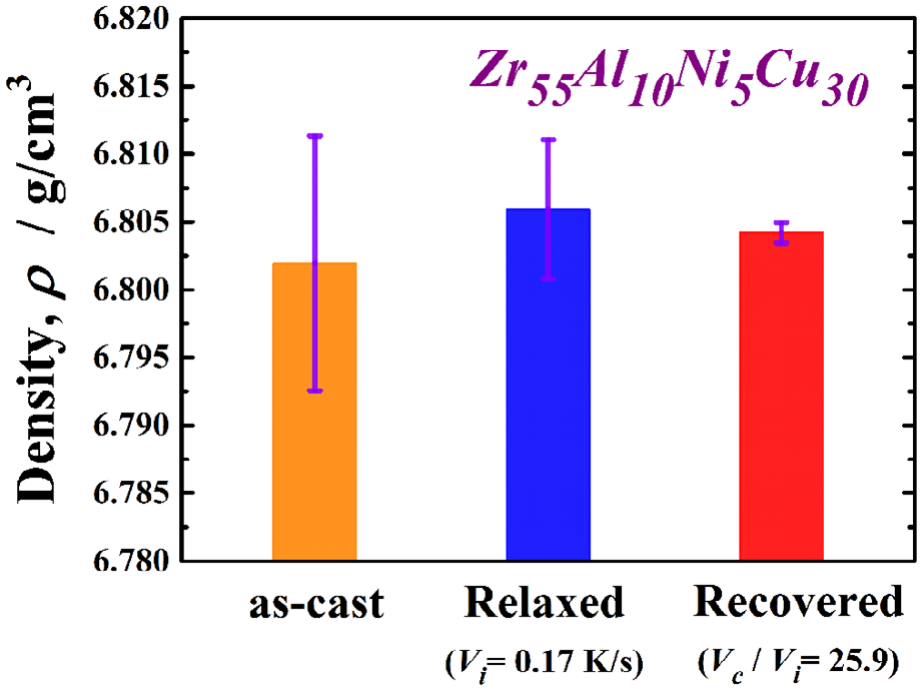
Density of the Zr_55_Al_10_Ni_5_Cu_30_ metallic glass in the as-cast, as-relaxed and as recovered at *V*_c_/*V*_i_ = 25.9 states. Here, recovery annealing was also performed at *T*_a_/*T*_g_ = 1.07.

Figure 2 shows the XRD pattern (a) and high-resolution TEM image and selected-area electron diffraction pattern (b) of the recovery-annealed Zr_55_Al_10_Ni_5_Cu_30_ disc sample for a temperature *T*_a_/*T*_g_ of 1.07 and cooling rate *V*_c_/*V*_i_ of 20. The XRD profile contains only a halo pattern and shows no significant diffraction peaks corresponding to a crystalline phase. The high-resolution TEM image clearly reveals maze-like contrast, and no obvious ordered clusters and/or nanocrystalline particles are observed. Similar XRD patterns were also obtained under other conditions in this study. These results indicate that the monolithic glassy structure remains after the present recovery annealing. The specific heat curves of *C*_p,s_ and *C*_p,q_ are shown in Figure 3(a). *C*_p,q_ is represented for three different states of the relaxed and recovery annealed (*V*_c_/*V*_i_ = 10.0 and 25.9 at *T*_a_/*T*_g_ = 1.07) samples. *C*_p,s_ and *C*_p,q_ are almost the same in the relaxed state, indicating that the sample reaches thermodynamic equilibrium after relaxation annealing. The curves of the two specific heats differ slightly in the higher-temperature region around 700 K because some non-relaxed glassy structure exists even after relaxation annealing at a slightly lower annealing temperature (685 K). However, *C*_p,q_ is clearly lower than *C*_p,s_ for both recovery-annealed metallic glasses. This result indicates thermodynamic instability, which is actually induced again by recovery annealing. Since the *C*_p,q_ curve at *V*_c_/*V*_i_ = 25.9 is lower than that at *V*_c_/*V*_i_ = 10.0, the recovery-annealed metallic glass with a higher cooling rate ratio, *V*_c_/*V*_i_, is in a more disordered (less relaxed) state. The change in ∆*H*_relax_ versus *V*_c_/*V*_i_ is plotted in Figure 3(b). ∆*H*_relax_ is approximately 0.76 J g^–1^ for *V*_c_/*V*_i_ = 2.0 and 2.56 J g^–1^ for *V*_c_/*V*_i_ = 5.0, which are almost equivalent to that for the relaxed state (1.42 J g^–1^). It significantly increases for values of *V*_c_/*V*_i_ over 10 and takes the maximum value of 9.74 J g^–1^ at *V*_c_/*V*_i_ = 25.9 under the present conditions. The degree of recovery is evaluated using $\Delta H_{\text{relax}}/\Delta H_{\text{relax}}^{\text{as - cast}}\;$, which is also shown in Figure 3(b). The recovery of the metallic glass samples depends strongly on the normalized cooling rate, *V*_c_/*V*_i_. Here, approximately 46% of the ∆*H*_relax_ corresponding to the as-cast glass can be recovered at the largest *V*_c_/*V*_i_ via a simple thermal treatment of low-temperature post annealing just above *T*_g_, followed by a suitable cooling process, even if the glass is almost fully relaxed. The maximum *V*_c_/*V*_i_ is approximately 26 owing to limitations of the present experimental apparatus; however, metallic glasses might be further thermally rejuvenated by applying a forced cooling process by improving the furnace. In order to confirm that rejuvenation occurred, the density of the recovery-annealed glass was measured. A less relaxed glass is known to have a lower density because of its larger amount of free volume and disordered structure. Figure 4 exhibits the density of the Zr_55_Al_10_Ni_5_Cu_30_ metallic glass in the as-cast, as-relaxed, and as-recovery-annealed at *V*_c_/*V*_i_ = 25.9 states. Here, recovery annealing was also performed at *T*_a_/*T*_g_ = 1.07. The density was increased by relaxation owing to annihilation of the free volume or the defects described above. The density change during relaxation is approximately 0.06%, which is consistent with previous report [8]. The metallic glass transitions into a slightly loose-packed state again after recovery annealing. The density of the recovery-annealed glass is approximately 0.03% larger than that in the as-cast state; this difference is almost 50% smaller than that between the as-cast and relaxed states. This tendency corresponds to the change in the relaxation enthalpy, as shown in Figure 3. It is well known that the free volume disappears because of structural relaxation with increasing temperature up to *T*_g_, whereas it is induced again in the supercooled liquid state [38–40]. This result is attributed to the introduction of free volume or defects, which are frozen in the subsequent rapid cooling process. The results also provide evidence of structural rejuvenation under the present annealing conditions.

### 3.2. Modeling of thermal rejuvenation using MD simulations

**Figure 5.**
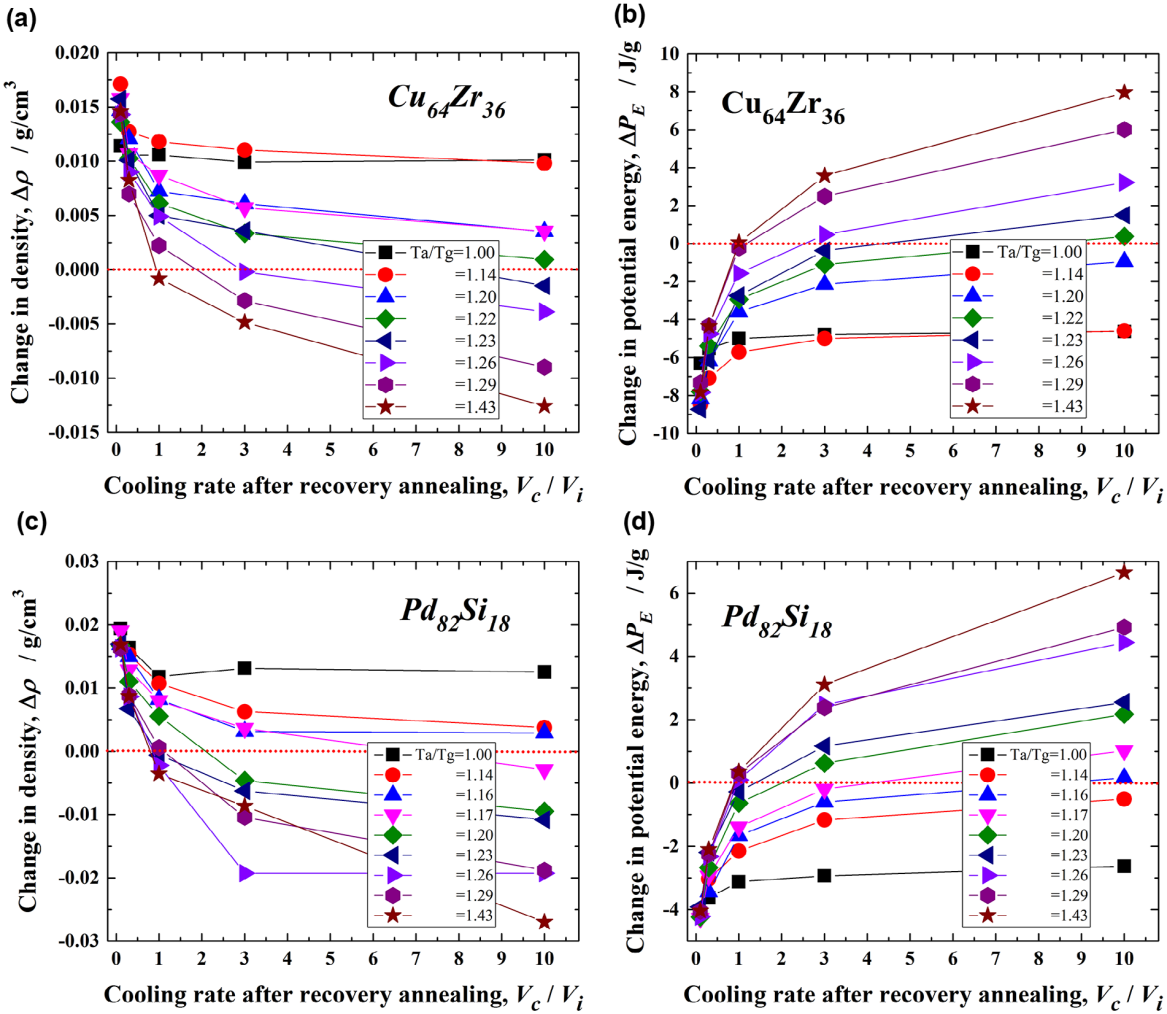
Changes in the density, Δ*ρ*, and potential energy Δ*P*_E_, with *V*_c_/*V*_i_ of the Cu_64_Zr_36_ (a and b) and Pd_82_Si_18_ (c and d) metallic glasses after recovery annealing at *T*_a_/*T*_g_ = 1.00–1.43.

Figure 5 shows the changes in the density, Δ*ρ* and the potential energy, Δ*P*_E_, with *V*_c_/*V*_i_ for the Cu_64_Zr_36_ (a and b) and Pd_82_Si_18_ (c and d) metallic glasses upon recovery annealing at various *T*_a_/*T*_g_ values between 1.00 and 1.43. The density generally decreases with increasing *T*_a_/*T*_g_ and also with increasing *V*_c_/*V*_i_ for both alloys. However, the condition of rejuvenation, which corresponds to Δ*ρ*<0, is limited. When the annealing temperature exceeds *T*_a_/*T*_g_ = 1.23 (~ 860 K) for Cu_64_Zr_36_ and 1.17 (~ 820 K) for Pd_82_Si_18_ at the maximum cooling rate ratio (*V*_c_/*V*_i_ = 10) in the present condition, the degree of rejuvenation is very low. In contrast, for larger *T*_a_/*T*_g_ values, such as 1.29 (~ 900 K) for Cu_64_Zr_36_ and 1.26 (~ 880 K) for Pd_82_Si_18_, rejuvenation occurs readily at a low *V*_c_/*V*_i_ of approximately 3.0. Moreover, no rejuvenation is observed for *V*_c_/*V*_i_ < 1.0, even if it is annealed at higher temperatures below *T*_a_/*T*_g_ = 1.43 (~ 1000 K). The potential energy increases in both alloys [Figure 5(b) and (d)] with elevating *T*_a_ and increasing *V*_c_/*V*_i_, which exhibits a similar tendency to the density change. That is, larger *T*_a_/*T*_g_ and *V*_c_/*V*_i_ values result in a positive energy change, which indicates that rejuvenation proceeds by annealing. The critical *T*_a_/*T*_g_ value for rejuvenation (Δ*P*_E_ > 0) for *V*_c_/*V*_i_ = 10.0 is 1.22 (~ 854 K) for Cu_64_Zr_36_ and 1.16 (~ 810 K) for Pd_82_Si_18_. Rejuvenation cannot occur at temperatures below these *T*_a_/*T*_g_ values. Moreover, *V*_c_/*V*_i_ > 3.0 is necessary for rejuvenation at *T*_a_/*T*_g_ > 1.26 (~ 880 K) in Cu_64_Zr_36_ and > 1.20 (~ 840 K) in Pd_82_Si_18_. These conditions for rejuvenation almost correspond to those for the density change. Thus, the potential energy is generally correlated with the density in metallic glasses, and those parameters are quite useful for evaluating the rejuvenation behavior. A comparison of the changes in the density and potential energy of these two metallic glasses indicates that rejuvenation would be easier in Pd_82_Si_18_ than in Cu_64_Zr_36_. For example, the critical *T*_a_/*T*_g_ for *V*_c_/*V*_i_ = 10.0 in Pd_82_Si_18_ is estimated at 1.17 (~ 820 K) according to the density change and 1.16 (~ 810 K) according to the potential energy change, and these values are lower than those [1.23 (~ 860 K) and 1.22 (~ 854 K), respectively] for Cu_64_Zr_36_. In addition, a higher cooling rate is necessary to induce rejuvenation in Cu_64_Zr_36_; that is, rejuvenation is achieved at *V*_c_/*V*_i_ > 2.5 according to the density change and at *V*_c_/*V*_i_ > 1 according to the potential energy change at *T*_a_/*T*_g_ = 1.29 (~ 900 K), and these values are larger than those (*V*_c_/*V*_i_ > 1 for both parameters) in Pd_82_Si_18_. The larger *V*_c_/*V*_i_ and *T*_a_/*T*_g_ demonstrate the difficulty of rejuvenation through the thermal process, suggesting that the rejuvenation behavior is associated with fragility.

## 4. Discussion

### 4.1. Change in mechanical properties owing to thermal rejuvenation

**Figure 6.**
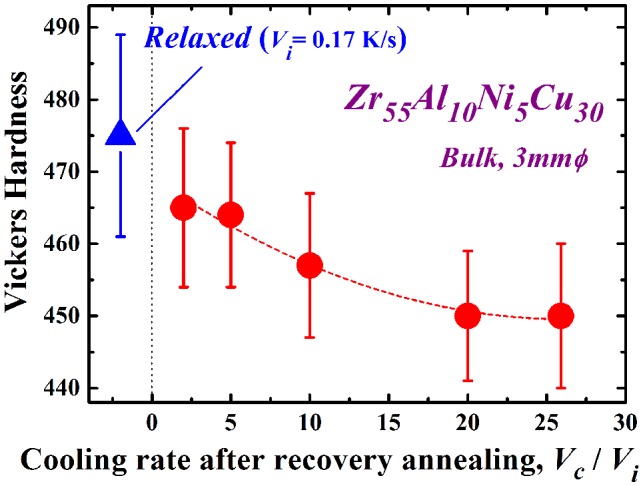
Change in the Vickers hardness *H*_v_ after recovery annealing at *T*_a_/*T*_g_ = 1.07 in the Zr_55_Al_10_Ni_5_Cu_30_ disc, which is re-plotted with *V*_c_/*V*_i_ in [32].

**Figure 7.**
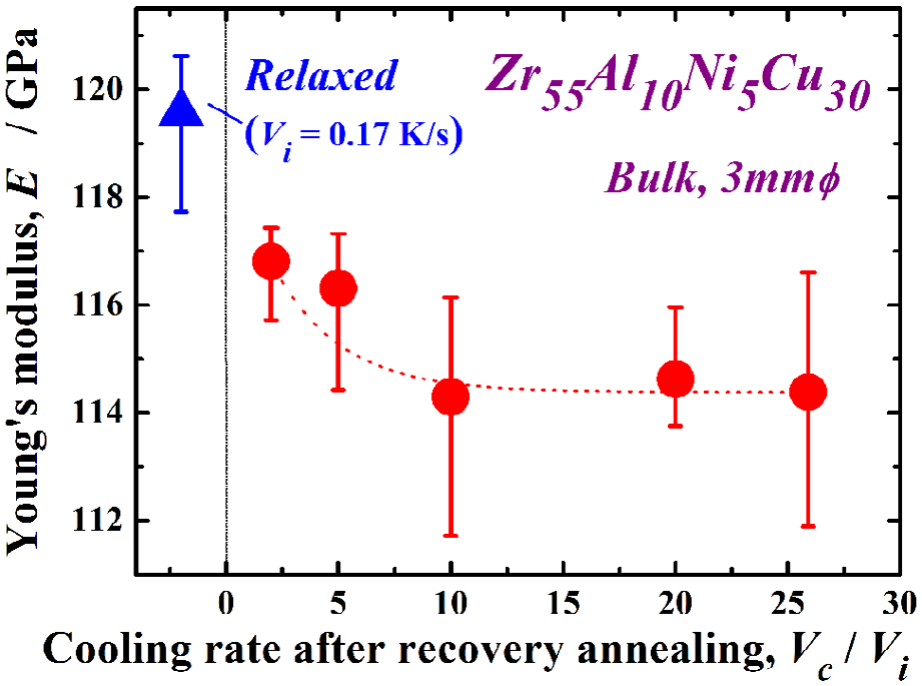
Change in the Young’s modulus *E* of the Zr_55_Al_10_Ni_5_Cu_30_ disc with *V*_c_/*V*_i_ after recovery annealing at *T*_a_/*T*_g_ = 1.07.

The mechanical properties of metallic glasses are known to depend strongly on their glassy structure. In particular, the relaxation state has a marked effect on their mechanical properties [4,41,42] .Thus, the mechanical properties would probably be changed by the present rejuvenation process. We have preliminarily reported mechanical softening due to rejuvenation [32]. Figure 6 shows the change in the Vickers hardness, *H*_v_ after recovery annealing at *T*_a_/*T*_g_ = 1.07 in the Zr_55_Al_10_Ni_5_Cu_30_ disc, which is re-plotted with *V*_c_/*V*_i_ in the above-mentioned study. The *H*_v_ value of the as-cast sample is approximately 455, and the glassy alloy hardens itself by relaxation (*H*_v_ ~ 475) due to annihilation of the free volume and/or local ordering. As *V*_c_/*V*_i_ increases during recovery annealing, significant softening occurs. The *H*_v_ value of the sample rejuvenated at *V*_c_/*V*_i_ = 25.9 is almost the same as that in the as-cast state, even though only 46% of the relaxation enthalpy is recovered. Further evaluation is necessary to explain this result. Such mechanical softening depending on the cooling rate in recovery annealing reveals the transition of the disordered state again due to the re-introduction of free volume or defects. It is suggested that the mechanical softening is sensitive to this re-introduction, which is one of the reasons for the significant recovery of hardness compared to the recovery of the relaxation enthalpy. The change in the Young’s modulus, *E*, exhibits a similar tendency, as shown in Figure 7. Here, the typical Young’s modulus of the as-cast glass is approximately 112 GPa. The Young’s modulus also increases with relaxation (*E* ~ 120 GPa); however, it is reduced by almost 5% (~ 114 GPa) at *V*_c_/*V*_i_ = 25.9. These results demonstrate the pronounced mechanical softening in the present recovery annealing, which is enhanced with increasing cooling rate ratio, *V*_c_/*V*_i_.

**Figure 8.**
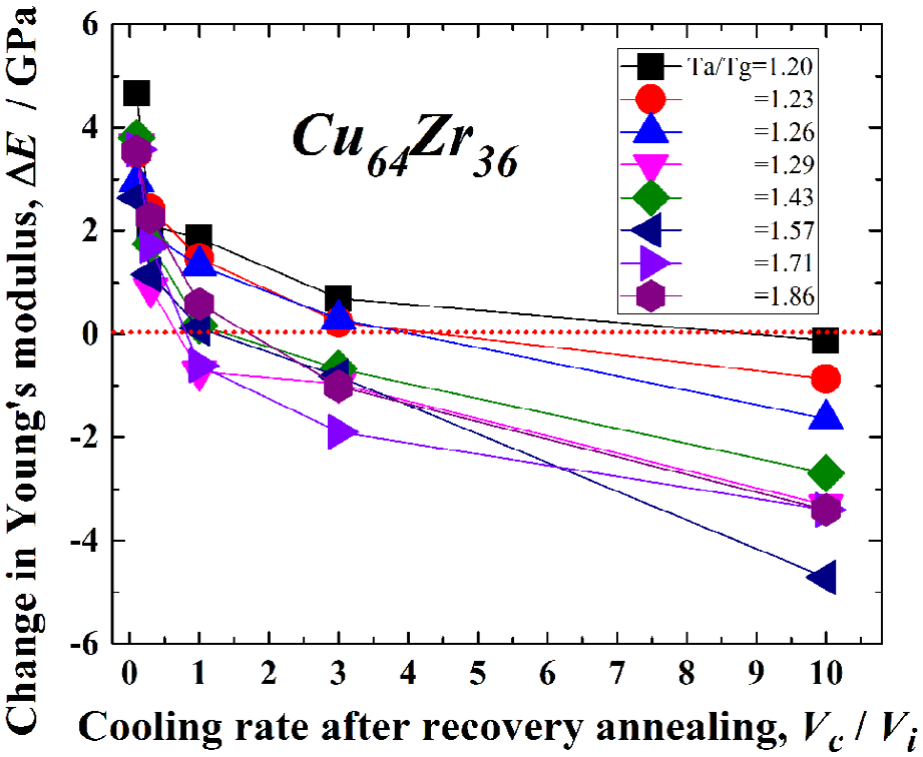
Change in the Young’s modulus, Δ*E*, with *V*_c_/*V*_i_ for *T*_a_/*T*_g_ = 1.20–1.86 (840–1300 K) in the Cu_64_Zr_36_ metallic glass.

The detailed conditions for mechanical softening are also examined using MD simulations of the Cu_64_Zr_36_ metallic glass. The change in the Young’s modulus, Δ*E*, with *V*_c_/*V*_i_ is shown for various *T*_a_/*T*_g_ values between 1.20 and 1.86 (840 and 1300 K) in Figure 8. Here, Δ*E* represents the difference of the Young’s modulus from that in the as-cast state. Mechanical hardening occurs in the relaxed state, i.e. for *V*_c_/*V*_i_ ~ 0, and it does not depend on *T*_a_/*T*_g_. The recovery-annealed metallic glass undergoes mechanical softening (i.e. a decrease in the Young’s modulus) with increasing *V*_c_/*V*_i_, especially at larger *V*_c_/*V*_i_ and *T*_a_/*T*_g_. The critical annealing temperature for rejuvenation (Δ*E* < 0) is *T*_a_/*T*_g_ = 1.20 at *V*_c_/*V*_i_ = 10. Moreover, the annealing conditions for which the Young’s modulus is lower than that in the as-cast state, are for *V*_c_/*V*_i_ > 3 and *T*_a_/*T*_g_ > 1.23. These conditions are obviously limited compared to those for rejuvenation evaluated from the density or potential energy change. Since it arises from macroscopic and cooperative dynamic manner during the mechanical softening process, the potential energy or density change, which is associated with structural changes at the atomic level, is a more suitable or direct index for evaluating the rejuvenation.

### 4.2. Rejuvenation map for metallic glasses

**Figure 9.**
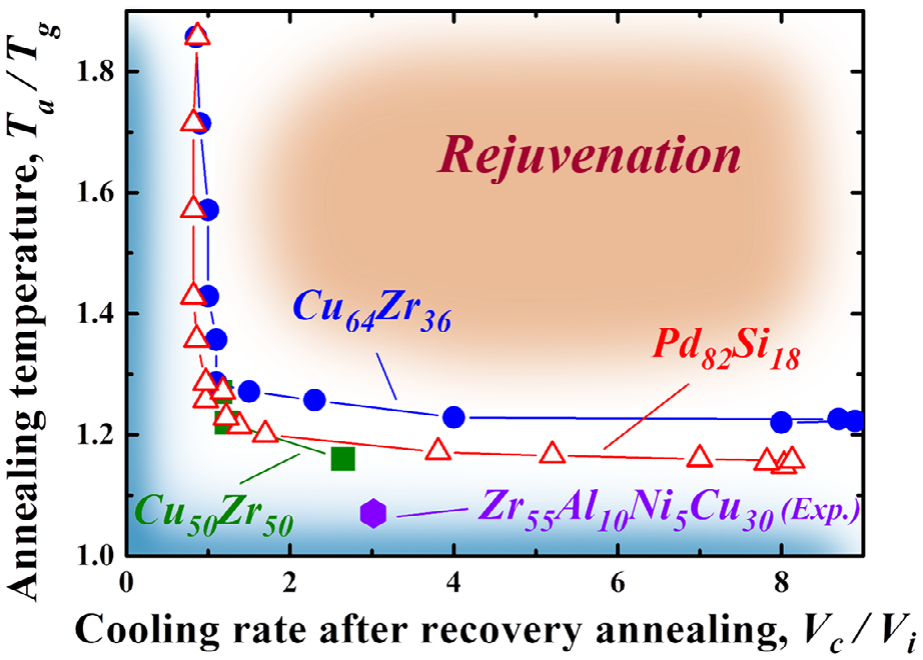
Rejuvenation map for the Cu_64_Zr_36_ and Pd_82_Si_18_ metallic glasses. Data for Cu_50_Zr_50_ from a previous simulation study based on the LJ potentials [33] and experimental data for the Zr_55_Al_10_Ni_5_Cu_30_ metallic glass are also presented.

As described above, the annealing temperature and cooling rate are considered to be the key parameters for rejuvenation. Metallic glasses must be in the supercooled liquid state preferably above ~1.2*T*_g_ for obvious rejuvenation, in which the relaxation history induced by thermal processes below *T*_g_ is erased, and a new liquid-like structure depending on the temperature is constructed. This was also predicted by a previous study [43]. Strictly speaking, metallic glasses must be held there for the duration of their relaxation time, *τ*. In this study, the holding time is fixed at 120 s in the experimental study and 2 ns in the MD simulations, and we do not investigate the influence of the holding time in recovery annealing. Since *τ* is experimentally estimated to be 143 s at temperatures close to *T*_g_ in Zr_55_Al_10_Ni_5_Cu_30_ [27], the present annealing temperature of 1.07*T*_g_ might be sufficient to form a new structure. Similarly, we can evaluate the validity of the annealing time used in the MD simulations. It is generally understood that the apparent *T*_g_ in MD simulations would be higher because the measurement time is shorter than that in experiments. The α-relaxation time is calculated as ~10 ns at *T*_g_ in Cu-Zr metallic glasses [44]. It decreases with elevating temperature according to the VFT (Vogel–Fulcher–Tammann) equation and is estimated to be less than 1 ns at approximately 1.07*T*_g_. Hence, the present conditions are also sufficient to realize the equilibrium state at the holding temperature. After the development of the new structure at *T*_a_, in which disordered structure as well as the excess free volume is induced, a new metallic glassy structure is formed depending on the subsequent cooling rate. Therefore, the cooling rate significantly affects the relaxation state after recovery annealing. Rejuvenation is evaluated by comparing the relaxation states before and after recovery annealing. Thus, the cooling rate ratio of the starting materials and the recovery-annealed glassy alloys, *V*_c_/*V*_i_, has to be monitored carefully. On the basis of these discussions, we can prepare a map of rejuvenation using the two parameters described above. Figure 9 shows the rejuvenation map for the Cu_64_Zr_36_ and Pd_82_Si_18_ metallic glasses evaluated by MD simulations. The map is evolved by the change in the potential energy. Data for Cu_50_Zr_50_ from a previous simulation study based on the Lennard-Jones (LJ) potentials [33] and experimental data for the Zr_55_Al_10_Ni_5_Cu_30_ metallic glass are also presented. Each plot displays the critical point for rejuvenation under the present conditions, in which higher *T*_a_/*T*_g_ values (upper side of the figure) and larger *V*_c_/*V*_i_ values (right hand side of the figure) correspond to the rejuvenated area (orange region). Rejuvenation is generally enhanced for larger *T*_a_/*T*_g_ and *V*_c_/*V*_i_. In contrast, the blue area indicates a more relaxed state. Regarding the annealing temperature, the critical point is located above *T*_a_/*T*_g_ = 1.2. The *T*_a_/*T*_g_ value of 1.2 is very important for resetting the thermal history and inducing a new glassy structure in metallic glasses, as noted previously. This critical temperature depends on the alloy composition. The Pd_82_Si_18_ alloy is easily rejuvenated a little bit as compared to Cu_64_Zr_36_, because the rejuvenation condition spreads into the lower *T*_a_/*T*_g_ side. In metal–metal type metallic glasses, it seems that we can rejuvenate Cu_50_Zr_50_ at a lower annealing temperature range than that for Cu_64_Zr_36_. These results probably originate from differences in the local structure, fragility, or potential used in the calculations; however, the reason should be investigated further. An experimental evaluation in the Zr_55_Al_10_Ni_5_Cu_30_ metallic glass exhibits a preferable tendency to undergo rejuvenation even at lower *T*_a_/*T*_g_ (~ 1.07), which suggests a correlation between the glass-forming and rejuvenation abilities of actual metallic glasses. We discuss this point further in the next paragraph. Additionally, these evaluations are based on the MD simulations, in which high heating and cooling rates of 1012–1013 K s^–1^ are assumed. In actual rejuvenation experiments on metallic glasses, rejuvenation and crystallization compete with each other, especially in the high-temperature region of ~*T*_a_/*T*_g_ ≥ 1.2, because the heating and cooling rates are comparatively slow, in the order of several kelvins per second. Finally, the cooling rate, *V*_c_/*V*_i_, does not exhibit such a clear composition dependence. This result indicates that rejuvenation could occur at a faster cooling rate than the initial one, i.e. *V*_c_/*V*_i_ ≥ 1, during annealing at an appropriate temperature [33]. The experimental results predict that disordered structure, which depends on the cooling rate, is formed after the thermal history is reset at approximately 1.2*T*_g_ in metallic glasses.

### 4.3. Mechanism of rejuvenation in terms of the local structure of metallic glasses

**Figure 10.**
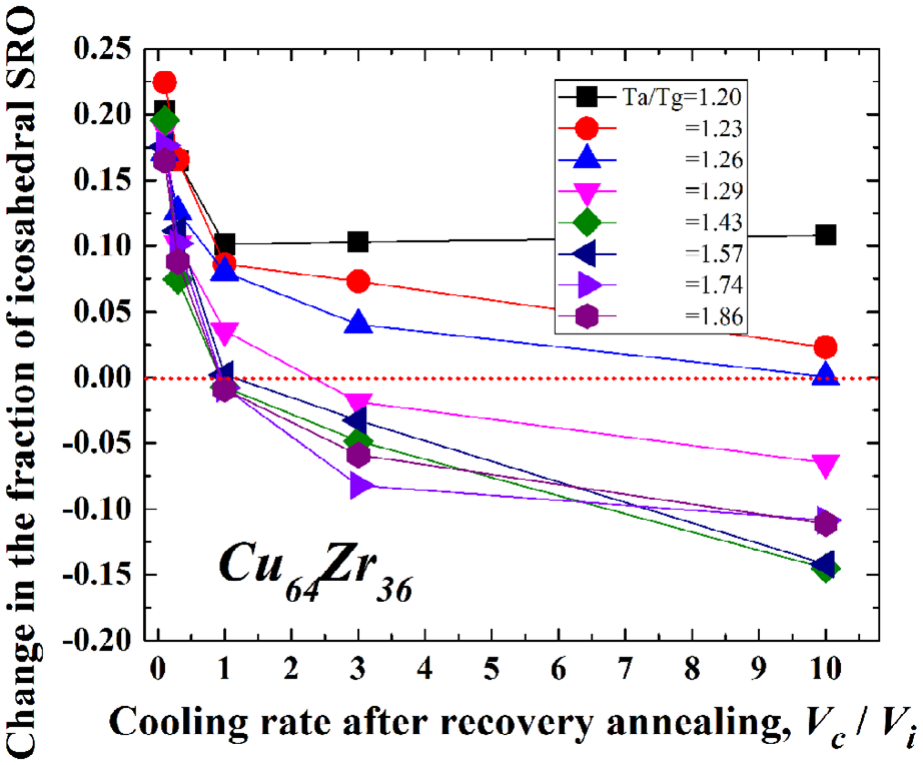
Change in the fraction of the icosahedral SRO in Cu_64_Zr_36_ with *V*_c_/*V*_i_ for *T*_a_/*T*_g_ ranging from 1.2 (~ 840 K) to 1.86 (~ 1300 K).

The relaxation state of metallic glasses strongly correlates with the local structure such as the free volume fraction, number of defects, or specific atomic configuration [45–47]. Thus, it is reasonable to apply this hypothesis to understand the rejuvenation behavior. Actually, the density change due to relaxation and rejuvenation, as shown in Figures 4 (experimental) and 5 (simulated), is roughly associated with the annihilation and re-introduction of free volume or defects. However, a change in the local atomic configuration, that is, a short- or medium-range ordered structure, as well as the free volume content, should also be considered because previous studies suggest that it contributes to the glassy nature [48–50]. Figure 10 shows the change in the fraction of icosahedral short-range order (SRO) in Cu_64_Zr_36_ with *V*_c_/*V*_i_ for various *T*_a_/*T*_g_ values ranging from 1.2 (~ 840 K) to 1.86 (~ 1300 K). The icosahedral SRO is correlated with the composition and is noticed as one of the key SROs to determine several properties of metallic glasses [46,51–53]. In the alloy, it might be formed mainly around the Cu atom [54]. Here, the fraction of the icosahedral SRO is normalized by that in the as-cast state; that is, a negative change in the fraction of the icosahedral SRO indicates rejuvenation. The icosahedral SRO is generated by relaxation and disappears with recovery annealing. Larger *V*_c_/*V*_i_ values (faster cooling in recovery annealing) and *T*_a_/*T*_g_ values (higher annealing temperatures) cause annihilation of the icosahedral SRO. The icosahedral SRO is recognized as being particularly sensitive to the cooling rate and relaxation history compared to other SROs [46,55]. Actually, the fraction of the icosahedral SRO depends remarkably on the cooling history, where less icosahedral SRO is formed at higher cooling rates or at elevated temperatures above *T*_g_.[46] The present results are highly consistent with these studies. During the recovery annealing process, the amount of icosahedral SRO increases initially up to *T*_g_ to reduce the configurational potential energy; however, it decreases drastically in the supercooled liquid state. As described above, the thermal history of metallic glasses is reset (erased) above 1.2*T*_g_, which is equivalent to the process of reduction in the amount of icosahedral SRO with respect to the local structure. It is, therefore, suggested that re-generation of the icosahedral SRO is suppressed when metallic glasses are cooled more rapidly. The critical *V*_c_/*V*_i_ value for rejuvenation is almost 10 at *T*_a_/*T*_g_ = 1.26; however, it drops drastically to around 1.0 at *T*_a_/*T*_g_ = 1.43, which reveals that the annealing temperature has a pronounced effect on the change in the local structure in the supercooled liquid state. Therefore, it can be concluded that rejuvenation is accompanied by a local structural change, especially in the formation of icosahedral SRO. Very recently, Miyazaki et al. [56] predicted pressure-promoted thermal rejuvenation originating from enhanced local ordering on the basis of MD simulations. In that case, the amount of icosahedral SRO increases with rejuvenation, even when the metallic glass is in a higher energy state, which contradicts the present results. This might imply that it is necessary to consider other aspects in addition to the change in the local structure of the icosahedral SRO to understand the rejuvenation behavior completely.

**Figure 11.**
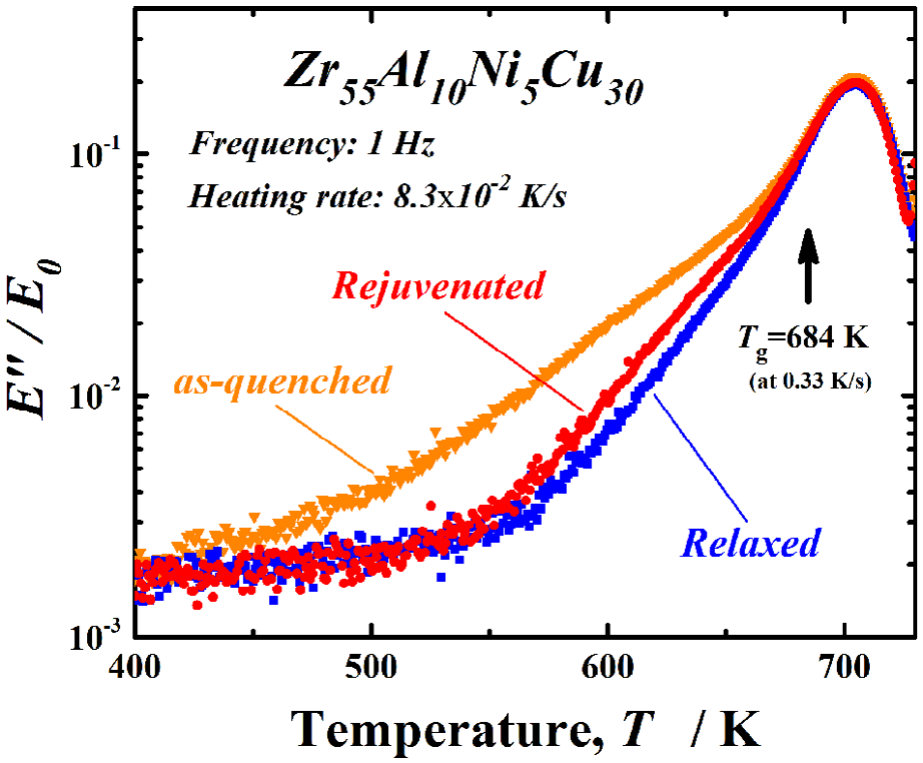
Internal losses in as-quenched, relaxed (annealed for 120 s at *T*_g_ and then cooled at 0.17 K s^–1^), and rejuvenated [relaxed sample annealed for 120 s at 1.07*T*_g_ (*T*_a_/*T*_g_ = 1.07) and then cooled at ~ 4.4 K s^–1^ (*V*_c_/*V*_i_ ~ 25.9)] Zr_55_Al_10_Ni_5_Cu_30_ metallic glass ribbon samples.

Finally, experimental rejuvenation in an actual metallic glass is investigated. As stated in Section 3.1, an almost fully relaxed Zr-based metallic glass disc could be thermally rejuvenated, and approximately 46% of the ∆*H*_relax_ value corresponding to the as-cast state could be recovered. Note that partial rejuvenation was achieved even at a low temperature of 1.07*T*_g_. In such cases, what is the primary location of rejuvenation in a metallic glass? To observe partial rejuvenation at low temperatures, tensile DMA was performed. Figure 11 displays the internal losses in as-quenched, relaxed (annealed for 120 s at *T*_g_ and then cooled at 0.17 K s^–1^), and rejuvenated [relaxed sample annealed for 120 s at 1.07*T*_g_ (*T*_a_/*T*_g_ = 1.07) and then cooled at ~4.4 K s^–1^ (*V*_c_/*V*_i_ ~ 25.9)] Zr_55_Al_10_Ni_5_Cu_30_ metallic glassy ribbon samples. The curve for the internal loss of the as-quenched sample exhibits an excess wing corresponding to β-relaxation prior to α-relaxation (the glass transition). The excess wing is reduced by relaxation, which exhibits the β-relaxation proceeds by this thermal process [57]. In the rejuvenated sample, the excess wing is considerably recovered, especially at approximately 600 K, compared to the relaxed sample. The α-relaxation peaks of the three samples are almost the same. Since the heating rate is slow in the DMA measurement compared to the relaxation time for α-relaxation around *T*_g_, the relaxation states of these samples may be almost the same. That is, the effect of rejuvenation is not as apparent in α-relaxation, and the results might even suggest that recovery and/or relaxation annealing affect their structure. Further, it is very clear that rejuvenation occurs preferentially in the β-relaxation region. β-relaxation has been attributed mainly to local rearrangement of atoms and free volume in the weakly bonded region [4,58]. Owing to the high atomic mobility based on the low bonding energy in this region, rejuvenation by rearrangement of atoms could preferentially begin here rather than in the strongly bonded region. Considering that the volume fraction of the weakly bonded region is estimated to be 20% in Cu-(Zr or Hf)-Ti metallic glasses [59], a limited area might be affected by low temperature rejuvenation. However, because the change in the enthalpy or density during rejuvenation could not be quantitatively related to the volume fraction of the weakly bonded region, it is necessary to investigate these points in the future. Furthermore, due to an improved inhomogeneous structure in metallic glasses with high glass-forming ability, they could have the advantage for rejuvenation by a thermal process. Finally, it is reported that mechanical rejuvenation using shot-peening, for example, also brings a strong effect on β-relaxation, accompanied by an increase in plasticity in metallic glasses [60], which leads to the important subject of the correlation between low-temperature relaxation and rejuvenation for improving the mechanical properties of metallic glasses intrinsically.

We conclude that partial rejuvenation at a low annealing temperature in metallic glassy alloys is particularly associated with atomic disordering in the β-relaxation region. The influence of rejuvenation on α-relaxation is not clear in the present evaluation. However, because the cooling rate around *T*_g_ is different among the as-quenched, relaxed, and rejuvenated samples, the glassy structure should be changed by this thermal treatment.

## 5. Conclusions

We investigate the structural rejuvenation in metallic glasses by a simple thermal process based on experimental and simulation studies performed for various alloy compositions. Rejuvenation by a thermal process is clearly confirmed by both approaches. Specifically, an increase in the potential energy, a decrease in the density, a change in the local structure, and mechanical softening are observed after thermal rejuvenation. Two parameters, the annealing temperature, *T*_a_/*T*_g_, and the cooling rate for recovery annealing, *V*_c_/*V*_i_, are quite important for evaluating the rejuvenation behavior. We successfully construct a rejuvenation map using these two parameters by MD simulations. In particular, because the thermal history of metallic glasses is reset above 1.2*T*_g_, which correlates to a change in the local structure, we conclude that the annealing condition of *T*_a_/*T*_g_ ≥ 1.2 is essential for ensuring rejuvenation. In this temperature range, the icosahedral SRO decomposes and the structure approaches that in the liquid state. It is, therefore, suggested that a new glassy structure is induced after cooling from the above-mentioned region, depending on the quenching rate. This is the dominant reason for thermal rejuvenation. Partial rejuvenation also occurs in the Zr_55_Al_10_Ni_5_Cu_30_ metallic glass after annealing at a low temperature of *T*_a_/*T*_g_ ~ 1.07 followed by rapid cooling. It originates from the inhomogeneous structure of metallic glasses with a high glass-forming ability. Since the weakly bonded (loosely packed) region may contribute to the preliminary atomic rearrangement in the disordered state, this region could be rejuvenated even at a low annealing temperature. This study provides new information on the mechanism and evaluation of rejuvenation and new possibilities for controlling the structure of metallic glasses to improve their mechanical properties.

## Disclosure statement

No potential conflict of interest was reported by the authors.

## Funding

This work is supported by a Grant-in-Aid of the Ministry of Education, Sports, Culture, Science and Technology, Japan, Scientific Research (A) [Grant number 23246109] and by the ‘Promoted Program for Interdisciplinary Research’ of the Frontier Research Institute for Interdisciplinary Sciences (FRIS), Tohoku University.
